# Suicide Risk and the Economic Crisis: An Exploratory Analysis of the Case of Milan

**DOI:** 10.1371/journal.pone.0166244

**Published:** 2016-12-29

**Authors:** Isabella Merzagora, Giulia Mugellini, Alberto Amadasi, Guido Travaini

**Affiliations:** 1 Sezione di Medicina Legale e delle Assicurazioni – Dipartimento di Scienze Biomediche per la Salute – Università degli Studi di Milano, Milano, Italy; 2 Università della Svizzera Italiana, Institute for Public Communication, Lugano, Switzerland; University of Toronto, CANADA

## Abstract

In the past five years, several scientific articles have claimed that the increase some countries have registered in suicide rates since 2008 is somehow related to the economic crisis. Other research has suggested that the impact of specific economic problems on the probability of suicidal behavior is often mediated by other individual-level factors, mainly psychological and physical, whose negative influence is exacerbated by reductions in the availability of health and social care during an economic crisis. On the basis of almost 1,000 cases of suicide collected by the Institute of Forensic Medicine in the province of Milan, this article aims at testing whether suicidal probability during an economic crisis is influenced by the interaction between an individual’s employment status and the presence of psychological or physical disease. Using a binary logistic regression model, this article demonstrates that the likelihood of suicide during an economic crisis is three times higher for persons affected by a severe disease, either physical or psychological, than for people who are not affected (OR = 3.156; 95% CI = 1.066–9.339; p = 0.38). Neither employment status nor the interaction between employment status and health status contributed to the difference between the suicide rate before and during the economic crisis.

## Introduction

In the past five years, several research reports [1, 2] and scientific articles [3, 4], have highlighted the increasing number of suicides linked to economic reasons since the economic crisis started in 2008.

According to Link Lab [2], in Italy the number of suicides linked to economic reasons increased to as much as 40% of the total during the last four months of 2013. This increase is particularly striking as this type of suicide accounted for 6.1% of the total in 2010, 6.6% in 2009 and 5.3% in 2008. At the same time, several scientific papers and international research [3, 4, 5, 6, 7] have claimed that the increase some countries have registered in suicides since 2008 is somehow related to the economic crisis.

Stuckler et al. [7] analyzed suicide trends in ten European countries and noted that in all but one the suicide rate increased between 2007 and 2009. Recent national studies in England [8], Italy [3] and the United States [9] also revealed significant increases in suicide rates between 2008 and 2010. An analysis of the relationship between suicides and unemployment in 27 European countries, demonstrated that there were 4900 extra suicides in 2009 compared with previous years (2000–2007) [5].

According to EURES [1] and De Vogli et al. [3], Italy has also witnessed an increase in suicide rate since the beginning of 2008, especially among men. Crevallo et al. [10] noted that in the Italian province of Turin the percentage of suicides linked to economic reasons doubled during the period of the economic crisis (2011–2013) in comparison with previous years (2002–2010).

All these studies claimed that the economic crisis somehow influenced—through the increase in the unemployment rate or prevalence of economic difficulties–the suicide rate in a way which cut across other macro and micro factors.

Differently, other international and national research suggests that during a period of economic crisis the impact of specific economic problems on the probability of suicide is often mediated by other individual-level factors, mainly psychological and physical, whose negative influence is exacerbated by reductions in the availability of health and social care [6, 11, 12]. Economic crises can, indeed, have a strong negative impact on the quality of social and health services because they often influence the public funding for these sectors. Many countries privatized health services, reduced staffing levels in the public sector and reduced public expenditure on social care and social assistance during the economic crisis and this created a situation of social inequality in which less wealthy people could not afford necessary medicines and services [11, 13, 14].

Social inequality worsened as a consequence of austerity policies, this was reflected at individual level in reduced wellbeing and increased incidence of anxiety and depression syndromes. This also contributed to an increase in chronic physical conditions, such as circulatory diseases, hypertension, strokes, etc. which are known to be influenced by stress [15, 16, 17, 18].

Countries where public investment in health services and public spending on social care was maintained, such as Iceland, Finland, Sweden and Germany, did not experience these problems during the economic crisis [11]. Uutela [19] noted that the effects of the economic crisis on the psychological health of the population were less serious in those countries where both formal and informal social networks remained solid and easily accessible.

The above presentation of evidence is not intended to minimize the negative effect of economic crises on suicides; rather to emphasize that their impact on the suicide is not solely attributable to the worsening of individuals’ economic conditions.

The recent World Health Organization [12] report on prevention of suicide demonstrated that the interaction between biological, psychological, social, environmental and cultural factors has a significant influence on the variation in suicide rate across countries [12].

McLean et al. [20] classified factors contributing to risk of suicide into two main categories: 1) societal (i.e. macro-level, structural) and 2) individual (i.e. micro-level, biological, psychological and behavioral). Based on a systematic review of risk and protective factors for suicide they [20] also identified a third group of determinants: psychosocial factors. These factors represent an interaction of behavioral and social factors; the influence of social factors on an individual state of mind and behavior [21]. Societal risk factors become psychosocial factors only if they influence individuals’ health [21]. Family structure, school environment and employment status are societal factors which can have psychosocial effects on individuals. Martikainen et al. [21] argued that unemployment “is not a psychosocial risk factor when the impact on the individual is limited access to income and material goods, it becomes a risk factor only when it impacts on feelings of self-esteem that then impact on the health of the individual through modified behavior or psychobiological processes” (20: 15). Employment status should be considered a psychosocial factor if it affects health by influencing behavior and psychological or physical state at the individual level.

With this regard, several studies have suggested that unemployment can precipitate suicide, especially in interaction with psychological or physical illness, rather than being their main cause [22, 23, 24, 25, 26]. The WHO report [27] and other research on the effects of economic crisis, concluded that physical and psychological health are the individual variables which are most sensitive to economic changes [19, 28, 29, 30, 31]. In particular, according to the WHO [12] people suffering chronic pain or chronic disease have two to three times higher risk of suicide than the rest of the population. All illnesses associated with pain, physical disability, neurodevelopmental impairment and distress increase the risk of suicide (e.g. cancer, diabetes and HIV/AIDS) [12]. Preti and Miotto [32] investigated the trend in suicide rate during the economic crisis and they argued that psychological disease is the main predictor of suicide, although stressful events, such as losing one’s job, can act as enablers. In related research, Stuckler et al. [6] demonstrated that unemployment has a negative impact on psychological health, especially in the short-term.

According to Istat [33] 59.5% of suicides in Italy over the previous 10 years happened as a consequence of a psychological disease, 17.5% of a physical disease, 15.9% for sentimental reasons and only 6.3% for economic reasons. The presence of psychological or physical disease appears to be the main reason for suicide and together these motives accounted for more suicides (77%) than any other motive.

Losing one’s job, having difficulty paying for adequate housing and financial instability are all factors that can increase suicide risk in interaction with other issues, such as depression, anxiety, substance abuse, physical disease, difficult social relationships.

Based on this evidence the aim of this study was to test the hypothesis that the probability of suicidal behavior during an economic crisis is influenced by the interaction between an individual’s employment status and the presence of psychological or physical disease. To achieve this we analyzed data collected by the Institute of Forensic Medicine on suicides in the province of Milan. Milan is part of the region with the highest suicide rate in Italy [34]. Information about the relationships among the main individual and structural factors which influence suicide rate during an economic crisis provides vital evidence for determining public policy and identifying individuals at greater than average risk.

## Materials and Methods

This study addressed the following research questions:

1. Does employment status influence suicide risk at the individual level during an economic crisis?
2. Is individual suicide risk during an economic crisis influenced by the interaction between employment status and the presence of psychological or physical disease?

In order to answer these questions we compared the influence of these two factors (employment status and presence of psychological or physical disease) on suicide risk during the period of the economic crisis (2008–2013) and the period immediately preceding it (2002–2007). In order to test this relationship we used the chi-square test and binary logistic regression.

The analysis considers individual suicides in the province of Milan and benefits from data collected by the Institute of Forensic Medicine, University of Milan. This database has the advantage of containing detailed information about victim characteristics, including their socio-economic and medical background, as well as the characteristics of the suicide event.

### The dataset

The database of the Institute of Forensic Medicine, University of Milan is updated monthly and includes all deaths registered as suicides in autopsy reports by the Milan mortuary since 2001. The autopsy report includes several groups of variables: personal information on the victim (e.g. gender, age, residence, educational level, marital status, type of job, consumption of alcohol, tobacco and drugs, medical history, use of medicines), information on the suicide event (location, date, method etc.) and several other variables based on the autopsy (date of death, type of injury, site of injury, cause of death).

The database includes data on all suicides registered in the 91 municipalities under the authority of the Milanese public prosecutor.

The data recorded by the Institute of Forensic Medicine, University of Milan are consistent with the data collected by Istat through the ‘Survey on causes of death’, which is the main official source of data on suicides in Italy. Data from these two sources, classified by gender and age group, are 99% correlated for the years common to both datasets (2009–2012). This demonstrates the reliability and completeness of the information gathered by the Institute of Forensic Medicine, University of Milan.

The main contributions of this study are the analysis of individual-level data and the analysis of detailed information about the suicide event.

### Defining the dependent variable: Suicides in the province of Milan

Cases of suicide in Milan were divided into two categories, those which occurred in the period immediately preceding the economic crisis (2002–2007) and those which occurred during the economic crisis (2008–2013).

The date for the start of the economic crisis was defined by inspecting the trend in unemployment rate in Italy as a whole and Milan in particular. Unemployment rate is a clear and straightforward indicator of economic downturn and is often used in the scientific literature to define economic crisis [7, 4]. Gross domestic product (GDP) is also often used as an indication of a country’s economic status, but as GDP changes more slowly it is a less sensitive indicator of the temporal parameters of an economic crisis. In fact, the majority of European countries registered increases in GDP during the worse part of the economic crisis (between 2010 and 2011), with GDP only starting to decline in 2012 [28]. For these reasons we chose to use unemployment rate to define the start of the economic crisis in Italy.

**Fig 1:**
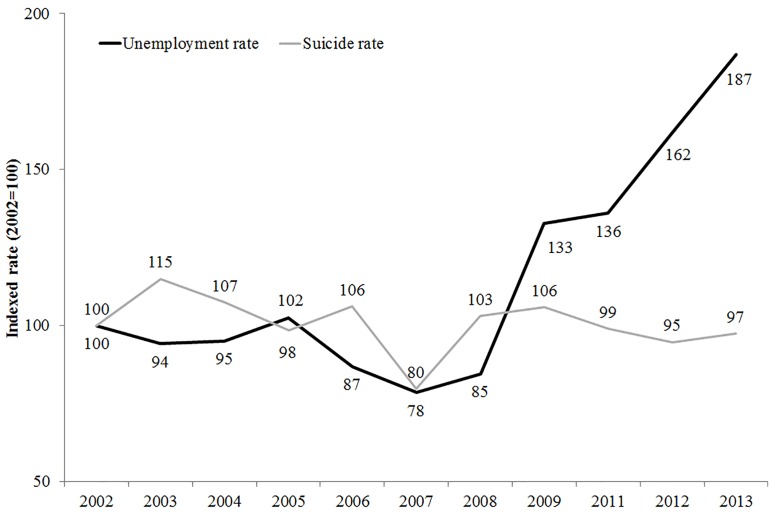
Trends in unemployment and suicide rates in the province of Milan. Index (2002 = 100). Years 2002–2013. Source: Authors, based on data from the Institute of Forensic Medicine, Milan and Istat.

Fig 1 shows that, in the province of Milan, unemployment started to increase in 2008 and continued to do so until 2013 [4, 7, 33]. This data justifies our decision to classify suicides occurring between 2008 and 2013 as having occurred during the economic crisis.

Our analysis categorized suicides according to whether or not they occurred during the economic crisis. This was represented as a dichotomous variable (0 for the period preceding the crisis, 2002–2007; 1 for the economic crisis, 2008–2013). Both periods were of equal length (six years).

Table 1 presents descriptive statistics for suicides occurring in the province of Milan between 2002 and 2013, based on data from the Institute of Forensic Medicine.

**Table 1:** Descriptive statistics on suicides in the province of Milan (2002–2013).

*Years*	*2002–2013*	*2002–2007*	*2008–2013*
**Total number of cases**	1893	933	969
**Annual mean**	158	156	160
**Standard Deviation**	14	17	11
**Maximum annual value**	174 (in 2003) 7 per 100,000 pop.	174 (in 2003) 7 per 100,000 pop.	173 (in 2013) 6.7 per 100,000 pop.
**Minimum annual value**	125 (in 2007) 5 per 100,000 ab.	125 (in 2007) 5 per 100,000 pop.	146 (in 2011) 5.8 per 100,000 pop
**Annual mean rate (per 100,000 pop.)**	6.2	6.2	6.2

Source: Authors, based on data from Institute of Forensic Medicine, Milan

The annual mean number of suicides in Milan is 158, which represents 6.2 per 100,000 population. The lowest rate during the period we analyzed was registered in 2007; the rate was highest in 2009 and in 2013.

**Fig 2:**
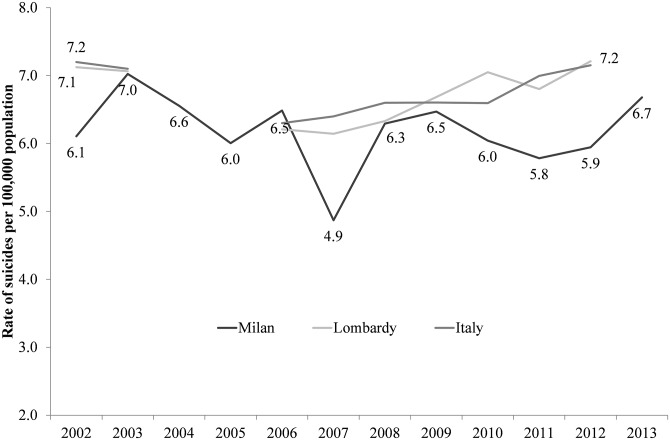
Number of suicides per 100,000 population in the province of Milan, the Lombardy region and Italy (2002–2013). Source: Authors, based on data from the Institute of Forensic Medicine, Milan and Istat.

As shown in Fig 2, the suicide rate in Lombardy (the Italian region of which Milan is the capital city) and Italy show a very similar trend. In Milan the average suicide rate was 6.2 per 100,000 between 2002 and 2013 whereas in Lombardy it was 6.7 per 100,000 and in Italy as a whole it was 6.8 per 100,000. The temporal trend in suicide rate in the province of Milan reveals that there is not a significant difference between suicide rate during the economic crisis (2008–2013) and in the period preceding it (2002–2007) (*Chi-square = 0*.*385*, *p = 0*.*535)*.

### Defining the independent variables: Employment status, psychological disease and physical disease

As already mentioned in the introduction, several studies highlighted that economic crisis should be considered a risk factor for suicides if it affects—through unemployment or other economy-related factors—specific individual problems, such as psychological or physical diseases. Drawing on this evidence, we decided to analyze the effects of three independent variables: employment status of the suicide victim (*employment status*), presence of physical or psychological disease (*disease*) and the interaction of these two variables (employment status*disease).

*Employment status* was represented as a dichotomous variable (0 = unemployed; 1 = employed). Cases where the victim was retired at the time of suicide were excluded from the analysis because it is believed that retired people are less affected by an economic crisis than those of working age and they might have psychological diseases related to senile dementia. Information about the victim’s employment status was collected at the time of autopsy by interviewing the victim’s partner, relatives or other persons close to him or her.

*Disease* was represented as a dichotomous variable capturing whether a suicide victim was or was not affected by physical or psychological disease at the time of suicide (0 = not affected; 1 = affected). For the purposes of this analysis, psychological disease was defined as psychological conditions such as anxiety and depressive syndromes, psychosis, schizophrenia, anorexia and other eating disorders. Drug dependency and alcohol dependency, which McLean et al. [20] and Rockville [35] considered to be among the main risk factors for suicide, were also considered among psychological diseases.

Physical disease included both chronic physical disease (e.g. diabetes, viral hepatitis, epilepsy, auto-immune diseases, etc.) and other serious diseases (e.g. cancer, cardiopathology etc.). Information about the presence of physical or psychological disease was obtained by the Institute of Forensic Medicine from inspection of the victim’s medical history and, where this was not available, by interviewing relatives, partner or other persons close to the victim, and from the results of toxicological tests.

We controlled for the influence of individual factors, such as gender, age, marital status (partnered; un-partnered) and residence context (urban; non-urban), on the relationship between the abovementioned variables and suicide risk.

Gender and age are the most important determinants of suicide risk. According to the WHO [12], men are 3.5 times more likely to be victim of suicide than women in developed countries and 1.6 times more likely to do so in developing countries. Data from Istat [32] confirmed that this pattern is observed in Italy: men have three times higher suicide risk than women. *Gender* was included in the analysis as a binary variable (0 = male; 1 = female). Istat [32] also found that the people older than 65 years were eight times more likely to commit suicide than people younger than 25 years. *Age* was represented as a binary variable (1 = victims aged between 25 and 34 years old; 0 = victims of all other ages). This classification was adopted on the grounds that people aged between 25–34 years are of working age and thus vulnerable to the effects of an economic crisis. The variable *marital status* distinguished between individuals who were married or cohabiting at the time of suicide (1) and individuals who were single, divorced or widowed (0). A systematic review [20] concluded that being married or living with a partner has a strong protective effect against the socio-economic risk factors for suicide.

The variable *urban context* was used to capture whether the suicide victim lived in the municipality of Milan (1) or outside the city (0). According to van Hooijdonk et al. [36] urban areas have a higher suicide rate than non-urban areas, mainly because of differences in population structure and the greater physical and social complexity of urban environments. However, the impact of living in an urban area is moderated by gender and age. Living in an urban area reduces the risk of suicide for young men but increases it for women [37].

## Results

The bivariate analysis reported in Table 2 shows that there was no relationship between employment status or health status and suicide risk during the economic crisis. However, it is important to note that more than 80% of suicide victims in the province of Milan between 2002 and 2013 were affected by a physical or psychological disease. This figure is in line with the data published by the WHO [12] and Istat [32].

**Table 2:** Correlation between suicides happened before and during the economic crisis, suicide victims’ employment status and health status.

Period of suicide	Employment status ^a^		Health status ^b^	
	*Unemployed*	*Employed*	*Disease present*	*Disease absent*
***Before the crisis (2002–2007)***	49.2%	50.2%	52.3%	49.5%
***During the crisis (2008–2013)***	50.8%	49.8%	47.7%	50.5%
**Total**	191	825	197	790

Source: Authors, based on data from the Institute of Forensic Medicine, Milan

^*a*^ *Chi-square* = .*058; p* = .810

^*b*^ *Chi-square* = .*491; p* = .483

The control variables age and marital status were negatively correlated with suicide risk during the economic crisis (Table 3). In the province of Milan people aged between 25 and 34 years had a lower suicide risk during the economic crisis than those in other age categories. Similarly, married and cohabiting couples also had lower suicide risk during the economic crisis than those who were unpartnered.

**Table 3:** Correlation between suicides happened before and during the economic crisis, suicide victims’ age and marital status.

Period of suicide	Age ^a^		Marital status ^b^	
	*25–34 years*	*Other age group*	*Married or cohabiting*	*Divorced*, *widowed or single*
***Before the crisis (2002–2007)***	56.2%	48.5%	61.7%	47.2%
***During the crisis (2008–2013)***	43.8%	51.5%	38.3%	52.8%
**Total**	203	813	196	820

Source: Authors, based on data from the Institute of Forensic Medicine, Milan

^*a*^ *Chi-square = 3*.*848; p ≤* .050

^*b*^ *Chi-square = 13*.*377; p* < .001

Binary logistic regression was performed to obtain a better understanding of how employment status affected suicide risk in the province of Milan during the economic downturn and to investigate whether the interaction between employment status and health status was an important factor in suicide risk. The binary logistic regression model included the two main independent variables (*employment status* and *health status*), another independent variable representing the interaction term for these variables (employment status * health status) and the control variables (*gender*, *age*, *marital status and urban context*).

The results of the binary logistic regression are summarized in Table 4.

**Table 4:** Binary logistic regression.

Independent variable *Period of suicide* (1 = 2008–2013, during economic crisis)	B	E.S.	Wald	Sig.	Exp(B)	95% CI exp(B)	
						Upper	Lower
*Gender (1 = Female)*	-.095	.155	.380	.538	.909	.672	1.231
*Marital status_(1 = Partnered)*	-.483	.175	7.596	.006	.617	.437	.870
*Urban context (1 = Urban)*	-.103	.144	.513	.474	.902	.680	1.196
*Age (1 = 25–34 years)*	-.396	.176	5.064	.024	.673	.477	.950
*Health status (1 = Disease present)*	1.149	.554	4.310	.038	3.156	1.066	9.339
*Employment status (1 = Employed)*	.729	.559	1.701	.192	2.073	.693	6.202
*Employment status by Health status*	-.889	.588	2.286	.131	.411	.130	1.301
*Constant*	-.734	.538	1.864	.172	.480		

Source: Authors, based on data from the Institute of Forensic Medicine, Milan. *Chi-square = 19*.*471*, *p* = .*007; -2 Log-Likelihood = 116*.*262; Cox & Snell R-Squared* = .*022; Nagelkerke’s R-Squared* = .*030*.

The results of the binary logistic regression demonstrate that, among suicide victims in the province of Milan, the likelihood of suicide during the economic crisis is three times higher for persons affected by a severe disease, either physical or psychological, than for people who were not affected. The presence of severe disease was a significant contributor to suicide risk during the economic crisis.

Neither employment status nor the interaction between employment status and health status contributed to the difference between the suicide rate before and during the economic crisis. Living with a partner can be considered a protective factor with respect to suicide risk during the economic crisis. The likelihood of suicide during the economic downturn compared to the pre-crisis period was 1.6 times lower for those who were married or cohabiting than for people who were divorced, widowed or single. Age also helped to account for suicide risk during the economic crisis. People aged 25–34 years old were 1.5 times less likely to commit suicide during the economic downturn than people in other age categories. Neither gender nor urban context contributed to the difference between the suicide rate during and before the economic crisis.

The binary logistic regression model was significant and accounted for 3% of the variance in suicide rate in the province of Milan. Sensitivity and specificity analysis is reported in Tables A-B and Fig A in S1 Annex.

## Discussion

Generally speaking, it is not easy to identify and quantify the effects of the economic crisis on suicide rate and health at population level, mainly due to the lack of up-to-date, reliable data. According to the WHO [12, 7] “since suicide is a sensitive issue, and even illegal in some countries, it is very likely that it is under-reported. Even in countries with good vital registration data, suicide may often be misclassified as an accident or another cause of death. Registering a suicide is a complicated procedure involving several different authorities, often including law enforcement. And in countries without reliable registration of deaths, suicides simply die uncounted”. For this reason official statistics often under-report suicide. In addition, suicide statistics very often do not include information about the method or other important characteristics of the event which are fundamental to developing effective prevention strategies.

Improving the availability and reliability of demographic statistics, public health statistics, and forensic institutes statistics, as well as developing sample surveys on the causes of death, is the prerequisite for developing effective suicide prevention programs [12]. The data collection protocol followed by the Institute of Forensic Medicine, Milan represents a good practice in this area. Their data covers a long time period (2001–2013) and is updated every month. It includes detailed information not only on the suicide event but also on the characteristics of the victims, including their socio-economic and health status. This database is useful for sociological research as well as forensic analyses. It enables the sociological researcher to analyze patterns in the main variables relevant to suicidal events, from the method used, to the location and the characteristics of the victims.

It is important to note that the analyses reported in this paper are exploratory and have some weak points. The main weakness relates to the difficulty of obtaining data and the problems inherent in this type of information, which might limit the reliability of the statistical analysis.

Although there is a 99% correlation between the data collected by the Institute of Forensic Medicine, Milan and the Istat data, some variables—such as that on the employment and health status of victims—remains problematic. This information is usually collected from pre-existing clinical documentation on the victim or, if this documentation is not available, by questioning relatives of the victim or other people close to him or her.

Preti and Miotto [32] noted that official statistics on the employment status of suicide victims might be biased. Relatives, and perhaps even the public authorities, might be more inclined to cover up the suicide of people in employment than unemployed people, for various reasons. Relatives might use the victim’s lack of employment as a justification for an event they cannot otherwise explain. Other people might be influenced by public opinion and the media, who often use unemployment and the economic crisis as scapegoats for these tragic events.

These types of bias could explain why employment status appears to influence suicide risk and should be borne in mind when considering evidence on trends in suicide.

In addition to these factors, the validity of the analysis of the relationship between suicide and the economic crisis in the province of Milan is limited by the failure to control for the influence of contextual variables such as investments in health and social care and income distribution, for which micro/individual level data are not available.

Given all these points, extreme caution should be exercised with respect to claims that of a causal relationship between economic crisis and suicides. This is true not only for the analyses included in this paper but also for the information provided by the media to the public.

These shortcomings notwithstanding this exploratory analysis may serve as a good starting point for similar research in other Italian provinces or at national level.

It would also be interesting, and in line with the recommendations of the WHO [12], to use data collected by all the forensic institutes in Italy in order to obtain a larger sample and hence more reliable statistical results. The information collected by these institutes should be well-suited to a pooled analysis because it is all based on standardized autopsy reports.

## Conclusions

The analysis of suicides in the province of Milan suggests that the relationship between the suicide rate and the economic crisis is mediated by individual factors other than unemployment, such as the presence of physical or psychological disease. In particular, this exploratory analysis of the data for Milan indicated that structural economic issues had less influence on suicide risk in people who were in good psychological and physical health. Such people can probably rely on solid cognitive and emotional barriers against external threats. If these barriers are weakened by severe psychological or physical disease, or by the lack of a stable emotional life (being without a partner), people become more vulnerable to structural economic pressures. This pressure, often worsened by the media, can instill feelings of social distress and insecurity.

Interestingly, the lack of an interaction between health and employment status suggests that suffering from severe disease during a period of economic crisis has a strong impact on suicide risk, independently from employment status. This may be due to the reduced availability and quality of health care during the economic crisis. The lack of effect of employment status may reflect the contemporary perspective on jobs. Jobs are regarded as more and more insecure and precarious and a job is viewed more as something with practical utility than as something with symbolic value that contributes to one’s identity. Other factors, such as age, health status and marital status contribute more to personal identity and are more strongly related to vulnerability to the negative effects of a socio-economic crisis.

In this context, it is important to emphasize that the economic crisis resulted in severe financial cuts to the public health sector and thus limited the availability of health care services. This may have exacerbated the difficulties faced by people affected by physical or psychological diseases, for example dealing with everyday stress or interpersonal problems [28].

The increase in suicide rate probably represents only the tip of the iceberg of health problems linked to the economic crisis. Several studies in various countries have demonstrated that the incidence of depression and anxiety syndromes and consumption of psychotropic drugs increase during periods of economic downturn [28].

The impact of austerity policies on the public health sector limited access to certain health care services and made them more expensive for patients (see Introduction). National governments, which are struggling to limit public expenditure, usually fail to meet the increased demand for health and social services which results from the increase in social distress and physical and psychological disease during an economic crisis. This results in a vicious circle, where the lack of proper health and social care during an economic crisis exacerbates social distress and health problems and thus increases the economic and human costs of the crisis.

The presence of a robust and efficient social and health care system is the key to limiting the negative impact of economic crisis on the psychological and physical health of a population. The WHO reported that the impact of an economic crisis on the health of a country’s population depends on action in five key areas: 1. active labor market programs; 2. family support programs; 3. control of the pricing and availability of alcohol; 4. debt relief programs; 5. primary care for the people at high risk of mental health problems [18].

This point further highlights the need, during a period of economic downturn, to identify population groups at high risk for suicide in order to focus scarce public resources on their needs. For this reason, research into suicide risk should be promoted and data on suicide rate and the factors which influence it, including psychological and physical health problems, should be widely disseminated.

## Supporting Information

S1 Annex Sensitivity and Specificity Analysis. (DOCX)
