# Supplementary material for: Suicide Risk and the Economic Crisis: An Exploratory Analysis of the Case of Milan
Source: PLoS One. 2016 Dec 29;11(12):e0166244. doi: 10.1371/journal.pone.0166244 (PMC5199046; doi:10.1371/journal.pone.0166244)
Supplement: S1 Annex — (DOCX) [file pone.0166244.s001.docx]

# Annex 1 – Sensitivity and Specificity Analysis

**S1 Table A. Case Processing Summary**

| **Period of suicide (1 = 2008-2013, during economic crisis)** | **Valid N (listwise)** |
| --- | --- |
| *Positive ^a^* | 419 |
| *Negative* | 438 |
| *Missing* | 1192 |

^a^ The positive actual state is 1.00.

**S1 Fig A. ROC Curve**

**
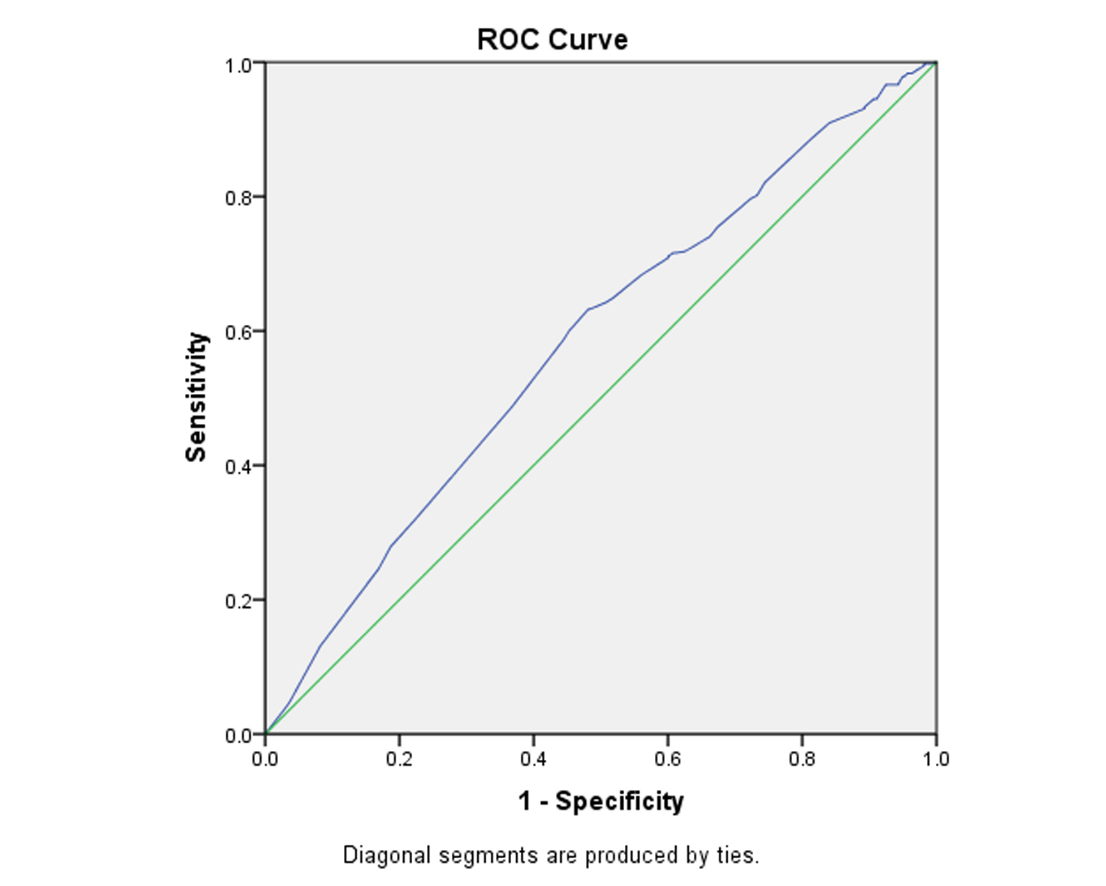
**

**S1 Table B. Area Under the Curve - Test Result Variable(s): Predicted probability**

| **Area** | **Std. Error ^a^** | **Asymptotic Sig. ^b^** | **Asymptotic 95% Confidence Interval** | |
| --- | --- | --- | --- | --- |
|  |  |  | **Lower Bound** | **Upper Bound** |
| .583 | .019 | .000 | .545 | .621 |

^a^ Under the non-parametric assumption

^b^ Null hypothesis: true area = 0.5
